# Prisoner’s dilemma game model Based on historical strategy information

**DOI:** 10.1038/s41598-022-26890-9

**Published:** 2023-01-02

**Authors:** Zhiqiang Gou, Ya Li

**Affiliations:** College of Computer and Information Science, Southwest University, Chongqing, 400715 China

**Keywords:** Computer science, Evolution

## Abstract

In many dilemmas, decisions are determined not by a single factor, but by multiple ones, including memory, reputation, reward and punishment. In recent years, how to design a mechanism to promote cooperation has become a research hot-spot. However, most of the previous studies mainly consider the historical benefits of the game, and pay less attention to the stability of the strategy (the frequency of strategy changes in the length of memory) and the proportion of memory in decision-making. The decision-making process of group evolution involves the influence of memory information on cooperative evolution in multi round games. It makes up for the lack of stability factors and weights in previous studies. Based on the above factors, a new strategy update rule is proposed to study the influence of the stability of historical strategy information on the evolution of cooperation in prisoner’s dilemma game, and the influence of memory weight on cooperation is considered. The stability of the current strategy is measured by the strategy in historical memory (the number of times the strategy in memory is continuous and consistent with the current strategy), which can determine the probability of an individual learning the neighbor strategy next time. Numerical simulation shows that an appropriate increase in the length of historical memory is more conducive to the emergence of cooperation, and the greater the weight of historical strategy information is, the more conducive to promoting cooperation, which shows that historical strategy information is still the main factor in decision-making. This study will help us understand the cooperative evolution of many real systems, such as nature, biology, society and so on, and effectively design reasonable mechanisms to promote cooperation.

## Introduction

According to Darwin’s view,“natural selection, survival of the fittest”, individuals are competitive^1^, and defection strategy is usually the optimal selection strategy in biological evolutionary game^2,3^. However, group cooperation still exists widely in real society. Therefore, how to understand the evolution of cooperation within selfish groups has become an active topic in social behavior science. How to improve the level of cooperation and what methods can be used to better cooperate have attracted great attention in the academic community^4–6^. Under such background, evolutionary game theory^7–9^ provides a good framework for explaining this phenomenon^10^, and results in many game models, such as prisoner’s dilemma game^11–13^, snowdrift game^14–16^ and ultimatum game^17,18^ and public goods game^19,20^. In addition, a considerable number of mechanisms such as space and network^21–23^ also play a very important role in promoting cooperation. In particular, Nowak and May^24^ created a milestone in spatial game and studied the cooperation between unrelated selfish participants. Evolutionary game has been widely studied in lattice network^25^ and complex network^26,27^. After Nowak and May, various mechanisms such as reward^28,29^, punishment^30^, practicality or weight^31^, personal mobility^32^, reputation mechanism^13,33–35^ have been added to spatial reciprocity to discuss whether cooperation can be further promoted^36–39^.

Individual memory is considered to be an important guide for future decision-making. Therefore, individuals may refer to their previous strategies in the future decision-making process in order to select the best strategy. At present, there have been a lot of studies on memory effect. Wang et al.^40,41^ proposed a snowdrift game model based on memory length. With the increase of payment parameters, the spatial mode and stepped cooperation frequency on the grid change. For scale-free networks, the high nodes occupied by collaborators lead to a high level of cooperation, and non-monotonic cooperative behavior is observed. Xia et al.^42,43^ proposed a game mechanism with memory on the regular lattice network. This mechanism only puts the benefits of the previous round of game into the current calculation without considering the evolution of multiple rounds of strategy states in the past. The individual’s memory ability, the proportion of memory and the size of the group will have an impact on the group evolution in the process of game. Zhu Hai et al.^44^ studied how to improve the emergence of heterogeneous policy update rules in collaborative scenarios, which has become an important open problem. The memory factor is introduced into the game model to study the joint effect of memory and heterogeneous strategy update rules on cooperation. Deng^45^ et al. proposed a historical optimal strategy learning mechanism considering historical strategy, income information and memory length, and combined them to discuss the generation and maintenance of cooperative behavior. The results show that the cooperation density is inversely proportional to the parameter *r* and positively correlated with the memory length *M*. Wang et al.^46^ proposed a mixed strategy prisoner’s dilemma game model. If individuals learn from neighbors who don’t often change their strategies, the whole team will be more cooperative. Different initial conditions have different evolution processes on the cooperation probability, but they will eventually reach the same cooperation level. Lu et al.^47^ studied the influence of memory effect on cooperative evolution in the spatial prisoner’s dilemma game, in which each player will record his strategy in the previous *M*-round game. The middle value of *M* will be most conducive to the emergence of cooperation. Challet and Zhang^48^ proposed a game model called “minor game” considering memory factors, in which individuals make decisions in the face of the game with the same information stored in their memory.

Based on inspiration of the above research, strategy updating is an important step in the process of evolutionary game, and the individual’s strategy updating is based on the current game of the individual or thinks that the individual only pays attention to the historical benefits when choosing the learning object. Zhu and Zhang^49,50^ put forward the so-called minority group game, that is, individuals can make decisions based on public information stored in memory. Previous studies have paid little attention to the stability of their own and neighbors’ historical strategies (i.e. the change frequency of strategies in memory length) and the proportion of historical strategy information in decision-making. The decision-making process of group evolution involves the effect of memory information on cooperative evolution in multiple rounds of games. It makes up for the lack of stability factors and weights in previous studies. When making decisions, individuals need to refer to their past strategic choices. In reality, individuals tend to learn from neighbors who do not change their strategies, because neighbors who do not change their strategies usually look more reliable.

Most of the previous studies mainly considered the historical payoff of the strategy updating stage, but paid little attention to the stability of the strategy (i.e. the frequency of the change in the memory length of the strategy) and the proportion of memory factors in the decision-making of the individual. This model makes up for the shortcomings of the stability factor of strategy information and the proportion factor of memory in previous studies. Based on the above factors, this model takes into account the level of strategy stability and the length of memory. In making decisions, individuals need to refer to past strategic choices. Individuals are more likely to learn from neighbors who stick to their strategy, because neighbors who do not change their strategy generally seem more reliable. Considering this, the parameter $n_{x}$ is introduced to represent the level of strategy stability of the individual, and the parameter *M* represents the length of historical memory. The memory in this paper refers to the number of rounds recorded by individuals. The memory length *M* represents that the individual has recorded the number of previous *M* rounds of games. That is, the individual will record the strategy of the previous *M* round game (cooperation or defection), and the next game, the individual will make decisions based on the strategy information. Considering that in many cases, individual behaviors and decisions are influenced by many factors, such as environment and emotion, the individual’s tendency to cooperate is not completely determined, so the parameter $\beta$ represents the weight of strategy information. The numerical simulation results show that the introduction of historical strategy information can promote the emergence of cooperation, that is, the appropriate increase in the length of historical memory is conducive to the emergence of cooperation, and the greater the weight of historical strategy information is, the more conducive to promote cooperation, which indicates that historical strategy information is still the main factor in decision-making.

The rest of the paper is organized as follows: Section “Results” describes the proposed model. Next, Section “Discussion” introduces the numerical simulation and analysis. Finally, in Section “Methods” we conclude with a summary.

## Results

Monte Carlo numerical simulation is used to simulate the evolution process of the whole population on a regular grid with $L \times L$ = $200 \times 200$ vertices. There are four neighbors around each individual, the initial cooperation probability is 50%, $K = 0.1$, and the system can be stable after $10 ^ {4}$ iteration.

**Figure 1:**
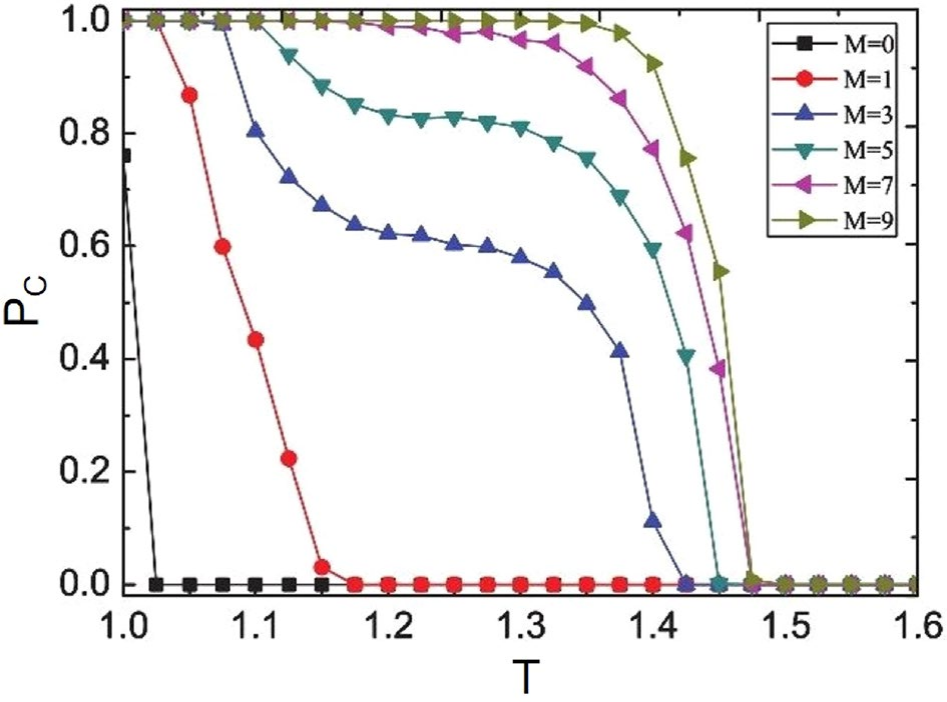
When $\beta$=0.5,$K = 0.1$, the evolution of cooperation rate with defect temptation *T* under different *M*($M=0, 1, 3, 5, 7, 9$).

In order to verify whether the introduction of historical strategy information can promote group cooperation. Firstly, the evolution trend of cooperation rate *Pc* with defect temptation *T* under different historical memory length *M* is explored, as shown in Fig. 1. It can be seen in the figure that when $M = 0$ (i.e. the traditional Fermi strategy update rule^49,52^), the cooperation of the group cannot be maintained, the strategy cannot promote cooperation, and the defector finally occupies the whole group. When $M > 0$ (i.e. considering the historical strategy information strategy update rules), the cooperation rate of the group gradually increases and the number of defects gradually decreases, especially when $M > 3$. For different historical memory length *M*, the cooperation rate of the group increases with the increase of *M*. When $M = 1$, because the individual has less historical strategy information, the individual has less information on the stability of the strategy and has less impact on cooperation. However, compared with $M = 0$, the threshold of defect temptation t increases. It can be seen from the figure that no matter how *M* increases, with the increase of *T*, when $T = 1.6$, the whole group is completely occupied by the defectors. Therefore, with the increase of historical memory information, individuals can better understand the strategic information of themselves and their neighbors, so as to make better decisions. If the neighbor has used the defect strategy before, it will not maintain high return, so the individual will not learn the low return neighbor strategy. Only through cooperation can we obtain high returns. Individuals are more inclined to learn the strategy of neighbors with high stability. Therefore, the consideration of historical strategy information can promote the cooperation level of the group. How to define the threshold of defect temptation to promote cooperation? Next, the relationship between cooperation rate and *M* under different defect temptation *T* is explored.

**Figure 2:**
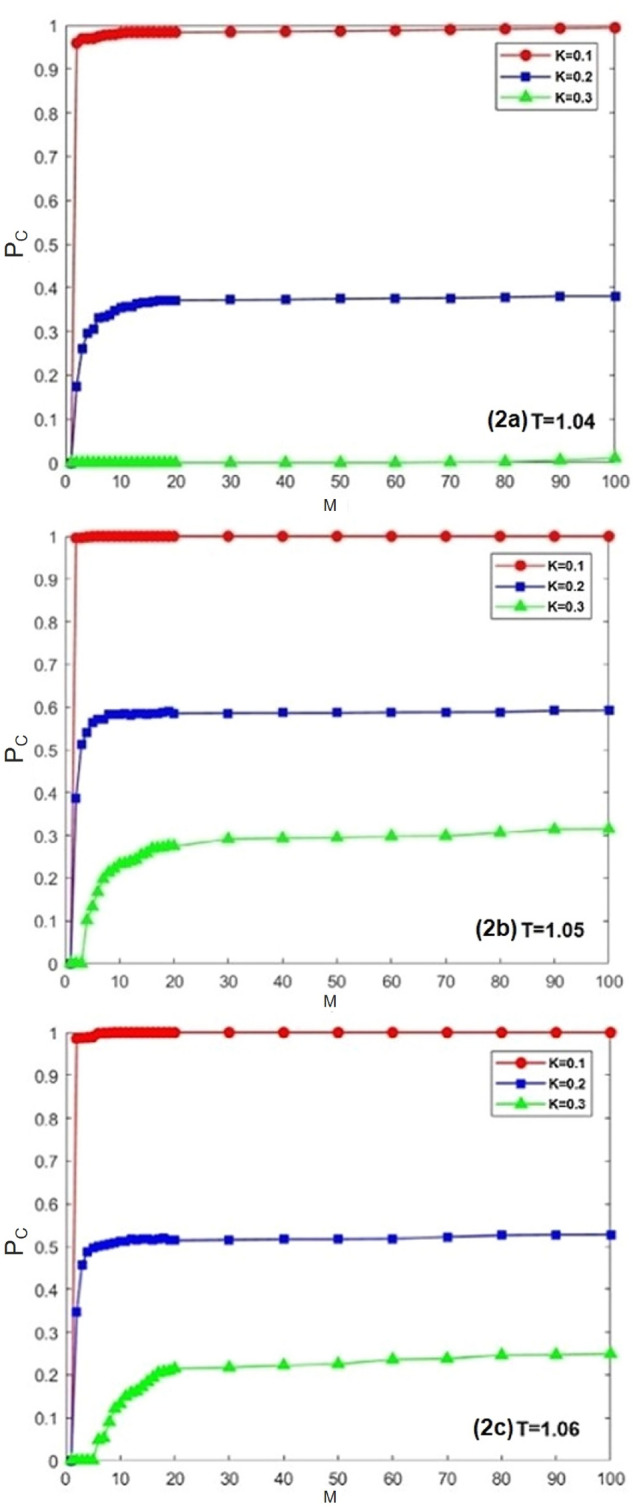
Evolution of cooperation rate with *M* under different *K* ($K = 0.1, 0.2, 0.3$) and *T*($T = 1.04, 1.05, 1.06$). The lattice size is set to be $L \times L$ = $200 \times 200$, $\beta$=0.5.

In order to better study the impact of memory length on group cooperation, as shown in the Fig. 2a–c are the simulation results obtained when the temptation factor *T* is 1.04, 1.05 and 1.06 respectively, while the red curve, blue curve and green curve in the figure represent the irrational impact on individuals in strategy selection, that is, the values of noise factor *K* are 0.1, 0.2 and 0.3. According to the simulation results in Fig. 2, the cooperation rate $P_{c}$ increases with the increase of memory length *M*. When the memory length *M* increases from 1 to 100, the cooperation rate $P_{c}$ will gradually reach the saturation state, and it can be seen from the figure that when the memory length reaches a certain length, the cooperation rate evolved in the group reaches the highest point for the first time, and then the cooperation rate evolved in the group remains basically unchanged with the increase of memory length. Therefore, it can be concluded that the introduction of memory effect in individual decision-making can effectively improve the cooperation rate within the group. It shows that the long memory length means that it can promote the evolution of the population. As described in the model, individuals may frequently change strategies by “resetting memory”, so as to make the cooperative behavior between individuals appear faster. But from the perspective of behavior, if this oppression is too great, it is not conducive to cooperation. In the public goods game, *M* will increase with the iteration of time, so as to promote the emergence of cooperation. In this study, *M* does not increase with time.

**Figure 3:**
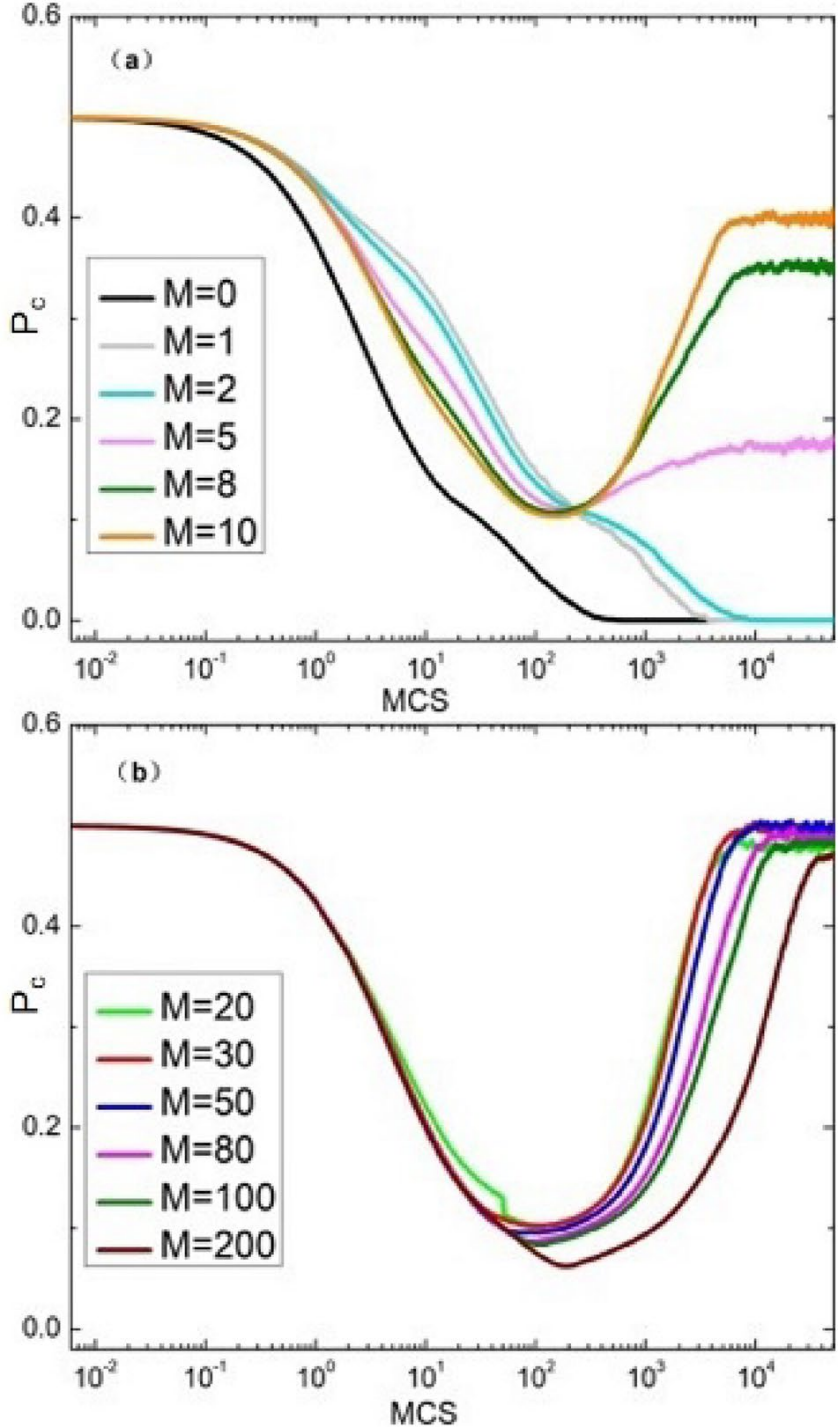
When $\beta$=0.5,$K = 0.1$, the evolution curve of cooperation rate with time under different *M*.

Secondly, in order to better explore the impact of the introduction of historical strategy information on group evolution. Fig. 3 depicts the evolution curve of cooperation rate with time under different *M*. As can be seen from the figure, the overall cooperation rate of the group first decreased and then increased. The decline of cooperation rate shows that the traditional space or network reciprocity^47,50^ does not meet the needs of group cooperation and cannot evolve cooperation. After a period of time, if the temptation of defects is not too great, you can create a cooperative cluster. However, under the current parameter setting, cooperative clusters cannot be extended under the traditional reciprocal conditions. Therefore, it is necessary to provide additional information and consider the historical strategy information to break this situation. Even if additional information is provided, the expansion of cooperation cannot be maintained due to the small amount of information. When the additional information provided is appropriate, this situation will change greatly. With the help of historical strategy information, the emergence and expansion of cooperative clusters can be maintained, so that the level of cooperation will gradually improve after about 100 time steps, as shown in Fig. 3. Because memory has a certain limit, too much information does not necessarily promote cooperation. Finally, continue to increase *M*, and the cooperation rate remains at the same level. The current research results show that the appropriate introduction of historical strategy information can support the generation of cooperative clusters.

**Figure 4:**
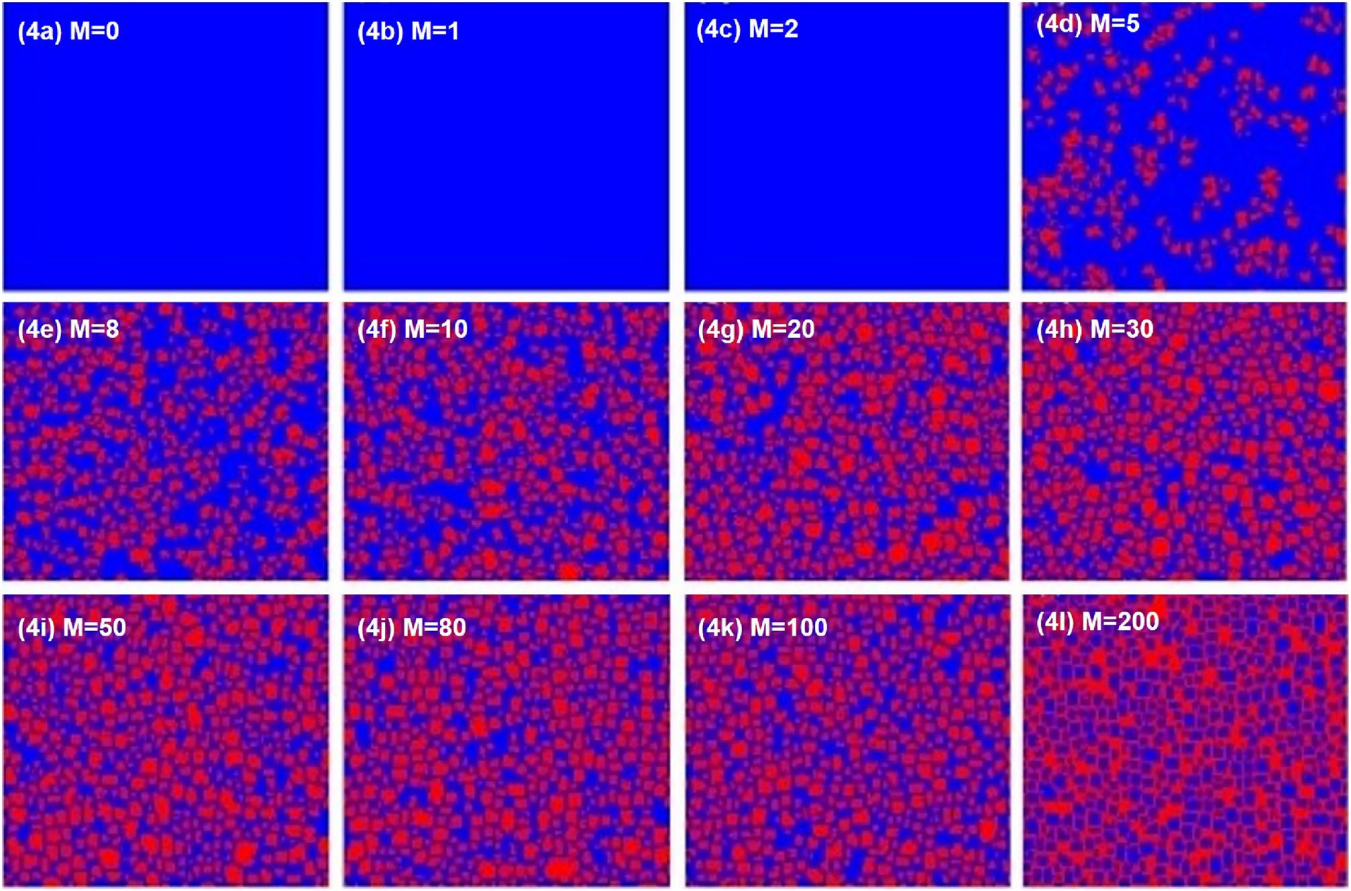
When $T=1.05$ and $K = 0.1$, the characteristic pattern of steady state under different *M* ($M = 0, 1, 2, 5, 8, 10, 20, 30, 50, 80, 100, 200$).

By observing the evolution curve of $P_{c}$ , it is considered that after introducing historical strategy information, partners can effectively resist the invasion of defectors by forming cooperation clusters. As shown in Fig. 4, when $T = 1.05$, it evolves to a stable state, and the distribution diagram of cooperators and defectors, in which red is cooperators and blue is defector. It can be seen from the figure that when the historical strategy information (i.e. $M = 0$) is not considered, the population finally evolves into traitors. When there is less historical strategy information (i.e. $M = 1$ or $M = 2$), the defector also occupies the whole population in the end. However, when there is more policy information ($M > 2$), some sporadic cooperative clusters begin to appear, such as Fig. 4d,e. After that, we can observe that as *M* increases from 10 to 30, the area of cooperative clusters further expands, as shown in Fig. 4f–h. In particular, when the historical strategy information is too large (i.e. $M > 30$), the cooperation cluster cannot continue to expand, and it is proved again that the introduction of historical strategy information can promote cooperation, but due to limited memory, more strategy information will not continue to promote cooperation, which may lead to the emergence of treason and will not create a better environment for the development of cooperation.

**Figure 5:**
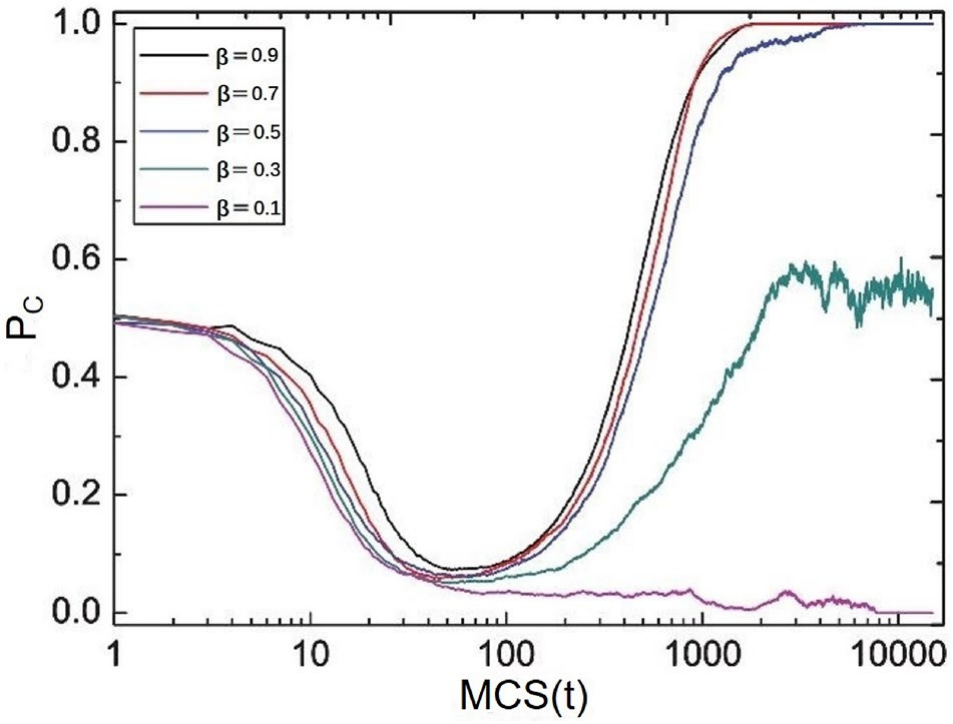
Evolution curve of cooperation rate with time at the stationary state for $T = 1.05$ under memory length $M=7$, $\beta$ is fixed to be 0.1, 0.3, 0.5, 0.7, 0.9.

**Figure 6:**
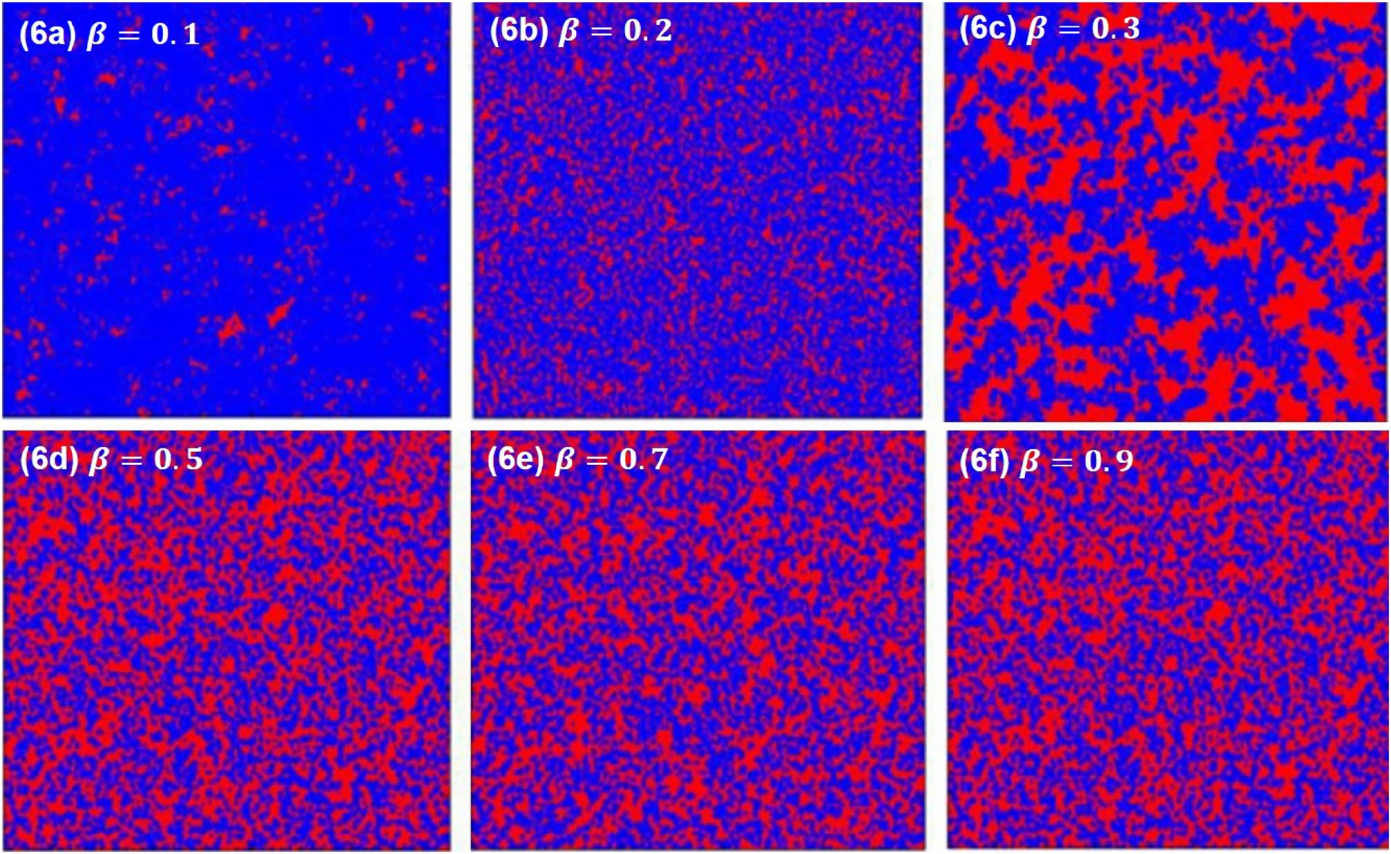
The characteristic diagrams at the stationary state for $T = 1.05$ under memory length $M=20$. From panel (**a–f**), the influence of memory weight $\beta$ is fixed to be 0.1, 0.2, 0.3, 0.5, 0.7, 0.9. The red and blue colors indicate “cooperators” and “defectors”.

Lastly, generally speaking, people’s historical memory information is limited. In order to objectively reflect this realistic factor and avoid disadvantages, the parameter of memory weight is introduced. Figures 5 and 6 obtain the heat map of collaborators and betrayers in steady state under different memory weights. From the above two parts, by observing the evolution curve of $P_{c}$, it is considered that after the introduction of memory effect, the cooperator can effectively resist the invasion of defector through the formation of cooperator clusters. A snapshot of the distribution characteristics of upper cooperators and defectors is obtained when the population evolves into a stable state, as shown in Fig. 6. From Fig. 6a–c, it can be found that when the degree of influence $\beta$ ( the values are 0.1, 0.2 and 0.3 ), almost all cooperators and defectors coexist in the stable state. With the increase of $\beta$, the number of cooperators is also increasing. From Fig. 6d,e that when the degree of influence $\beta$ ( the value is 0.4 or 0.5 ), there are only a few sporadic defectors, while most cooperators occupy the whole network. After that, with the increase of the $\beta$, the cooperator clusters expand further. In Fig. 6f, the cooperator clusters reach the maximum and will not continue to expand, and there are almost no traitors.The number of cooperators increases with $\beta$ increases. It is conducive to the emergence of cooperation and the improvement of cooperation efficiency. At the same time, it also proves once again that the long historical memory length do not brings more promotion to the group, which requires other conditions to jointly promote cooperation, which is conducive to the emergence of better cooperation. It also proves that in real life, when making decisions, people do not necessarily rely entirely on memory or experience, but the combination of more factors, and memory experience is only a part of it.

**Figure 7:**
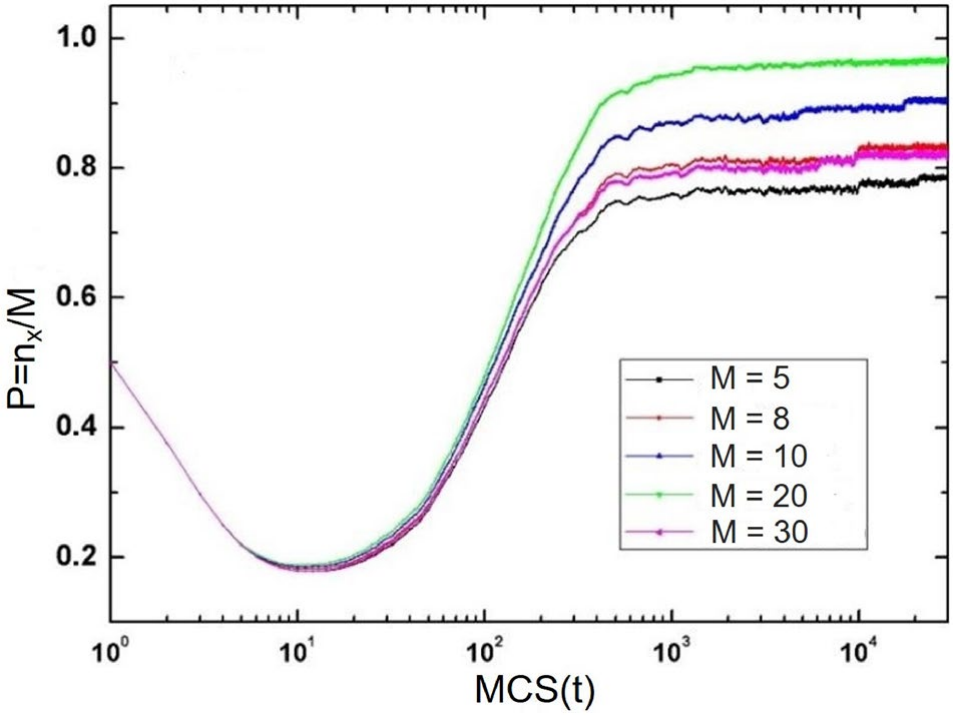
When $\beta =0.5$, the stability $\rho$ changes with time.

In order to further study the evolution trend of system cooperation frequency with time, we conducted simulation experiments on the change of strategy stability ($P = \frac{n_{x} }{M}$) with time under different historical memory length *M*. The experimental results are shown in the Fig. 7.

In the system, the strategy stability *P* decreases rapidly at the beginning, and gradually increases to a relatively stable value after reaching a minimum value. The minimum value of strategy stability *P* is 0.15, and the maximum stability of the system can reach 0.95 in the equilibrium state. In the mechanism based on historical strategy information proposed in this paper, when $M\>0$, the strategy stability of the system increases with the increase of *M*. Only when individuals properly adhere to the cooperation strategy can the cooperation frequency of the whole system reach the highest.

**Figure 8:**
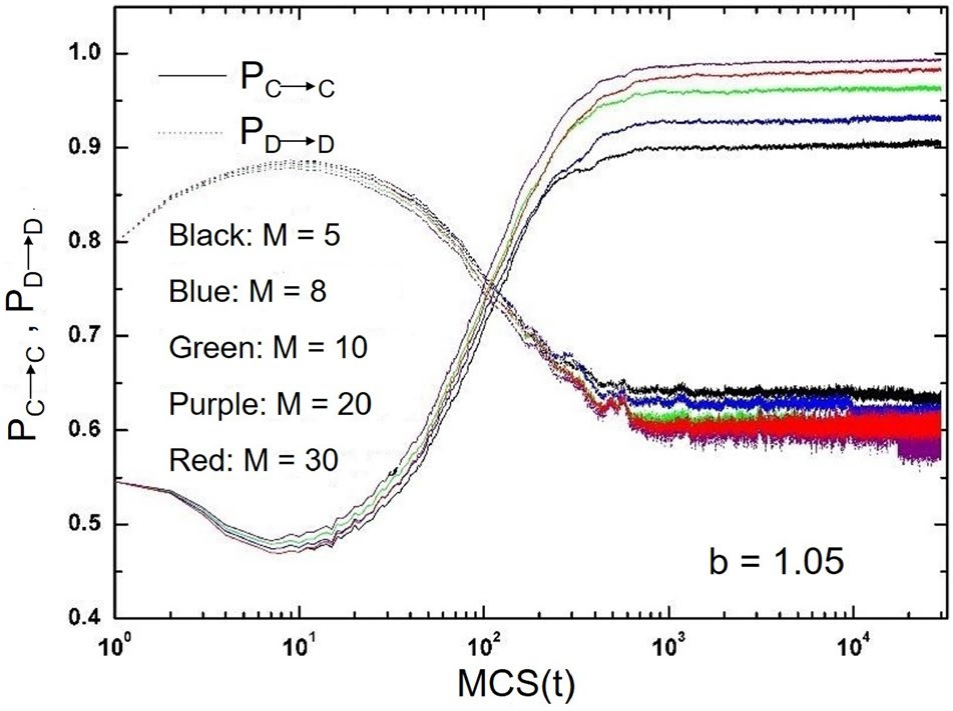
When $\beta =0.5$, the probability change of different strategies to remain stable.

The evolution of retention rates of two different strategies in the system with time is studied. Different strategy retention rates correspond to the stability of the whole system strategy, namely $\frac{n_{x} }{M}$. $n_{x}$ represents the same number of times as the current strategy. The greater the $n_{x}$, the greater the probability that an individual will keep the current strategy. $P_{C\longrightarrow C }$ represents the probability that the cooperator continues to maintain the cooperation strategy, while $P_{D \longrightarrow D }$ represents the probability that the defector continues to choose the defect strategy. It can be seen from the Fig. 8.

For different parameters *M*, the change trend of each probability is basically the same, but the change radian is different. At the beginning, the retention rate of the cooperation strategy is small, which is about 0.54, while the retention rate of the defect strategy is about 0.7. With the evolution, $P_{C\longrightarrow C }$ is decreasing while $P_{D\longrightarrow D }$ is increasing, which means that defect is the mainstream strategy of the system at this time. However, after some time, when the retention rate of the two different strategies is 0.75, the evolution has turned. At this time, $P_{C\longrightarrow C }$ will surpass PD, indicating that the cooperative strategy is gradually becoming a more dominant strategy. The bigger *M*, the earlier this turning point will occur. It can also be found from the figure that under the rules proposed in this paper, the cooperators will be more inclined to maintain the cooperation strategy, while most of the defectors will be inclined to choose the cooperation strategy and become the cooperators at the end of the evolution. These experimental results in this section are basically consistent with those in Fig. 3, which shows that the mechanism based on historical strategy information proposed in this paper can promote the emergence of system cooperative behavior.

**Figure 9:**
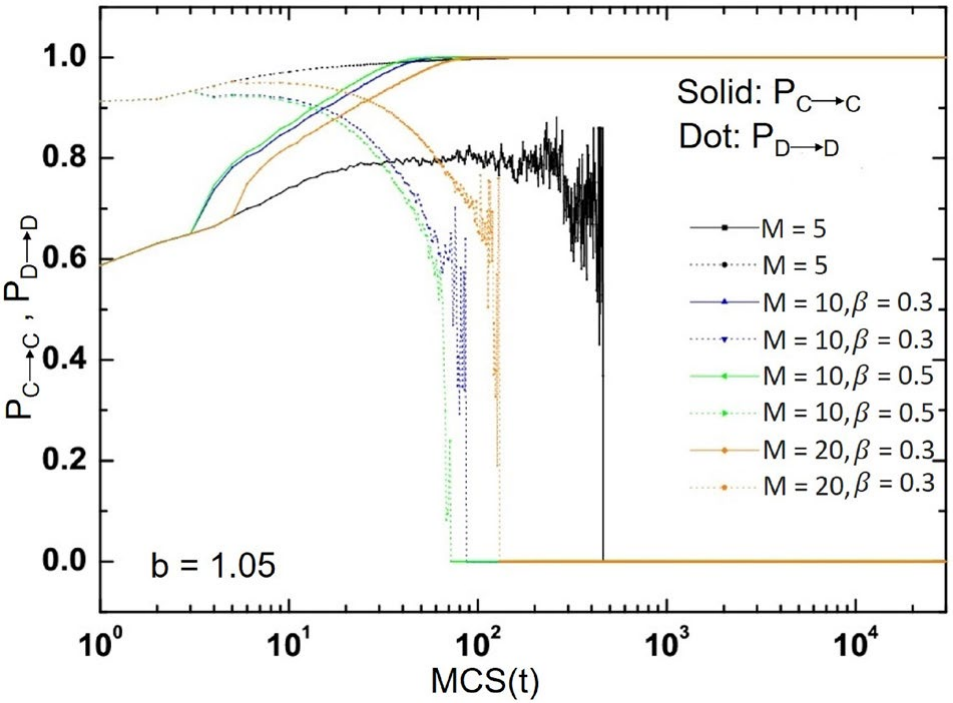
When $b=1.05$, the change of strategy stability with different *M* and $\beta$ values.

Finally, we also studied the evolution of the retention rate $P_{C\longrightarrow C }$ of cooperation strategy and the retention rate $P_{D\longrightarrow D }$ of defect strategy with time under different memory length *M* and memory proportion $\beta$ in the system, as shown in the Fig. 9.

$P_{C\longrightarrow C }$ represents the probability that the cooperator will continue to cooperate, and $P_{D \longrightarrow D }$ represents the probability that the defector will continue to choose the defect strategy. In the early stage of evolutionary game, $P_{C\longrightarrow C }$ will decrease and $P_{D\longrightarrow D }$ will increase, which means that the defect strategy is the mainstream strategy in the system at this time. However, after a period of evolution, $P_{C\longrightarrow C }$ will exceed $P_{D\longrightarrow D }$. It is worth noting that the greater the value of memory ratio $\beta$, the earlier the intersection of the two curves occurs. With the increase of *M*, the probability of cooperation strategy gradually increases. This means that the cooperators basically still choose cooperation strategies, on the contrary, most of the defectors will choose to adopt cooperation strategies at the end of the evolutionary game, thus becoming cooperators. Therefore, the numerical simulation results also show that the update mechanism based on historical strategy information is very effective in maintaining and promoting the cooperative behavior of the system.

## Discussion

Individuals have memory and memory is weighted, a prisoner’s dilemma game model based on historical strategy information is proposed in this paper. In the model, the evolution of group cooperation is studied by combining the historical strategy information and the change rate of memory weight. Numerical simulation results show that the change rate of historical strategy and memory weight can effectively promote cooperation. Larger memory length and larger memory weight are more conducive to promoting cooperation. This means that if individuals do not change strategies frequently, the whole group can promote cooperation. In the early stage of evolution, collaborators were invaded by a large number of defectors, but with the advancement of the evolution process, collaborators formed small clusters and gradually expanded. In addition, due to different initial conditions, different cooperation probabilities are formed in the evolution process, but the final level of evolutionary cooperation is the same.

At the same time, the research results of this paper are consistent with social phenomena, which can help us understand the cooperative evolution of many real systems such as nature, biology, society and engineering, and provide new ideas for promoting the design of cooperative mechanism. For example, in the social or financial system, credit scoring mechanism is used to promote everyone to keep their promises. The system records personal reputation information in the past period of time (reputation information can be expressed through memory effect). When individuals go to banks and other institutions to handle loans and other activities, the relevant departments can judge whether they can handle loans according to the credit information. This indirectly promotes everyone in society to be honest and trustworthy. In addition, the research shows that compared with the traditional policy update rules, the update rules considering historical policy information are more suitable to describe individual behavior. In the future work, historical strategy information and memory weight can be applied to other game models. At the same time, we can also explore the impact of different network topology on cooperation and the general scale of distress intensity.

## Methods

Prisoner’s dilemma game: if a prisoner chooses not to confess, this behavior is cooperation (C). For another prisoner, choosing to confess is defection (D). If two people cooperate, they can get their reward (reward, $R ( R = 1 )$). If both defect, they will be punished (punishment, P). If one person cooperates and one person defects, the cooperation gets “sacker’s payoff” (*S*), and the defector gets “betrayal temptation” ($T ( 1< T = b < 2 )$). Then *R*, *S*, *T* and *P* satisfy: $T\>R\>P\>S$ and $2R \>T + S$. The game is evolved on the $L*L$ grid network, that is, $N=L^{2}$. Each participant will be designated as a cooperation (C) or defector (D) with a 50% probability. In the process of population evolution, each participant plays games with its four nearest neighbors. Only the game model parameter t is considered here. In general, the weak prisoner’ dilemma model proposed by Nowak and May^51^ is adopted, where the parameter setup is set to be

1 $$\begin{aligned} R= 1,1< T\le 2,P= S= 0 \end{aligned}$$

Generally, without considering the parameter of memory information length, in order to maximize the benefits of the next round of game, participants update their strategies by comparing their own benefits with that of their neighbors. It is worth noting that no human participants were involved in the current study. This paper does not use human experiments and human tissue samples. We abstract the nodes in the network as individuals to play games.

Then, people have memories or experiences in real life and they often make decisions based on these memories or experiences. In order to introduce the historical memory information parameter, the right molecule of the above formula is replaced with the parameter $H_{x}$, which is related to the same strategy part as the current strategy in the previous *M* game rounds. In the previous *M* rounds of strategy update, individual *x* compares the current strategy with the previous strategy to make a decision. At the same time, the weight ($\beta$) of the parameter of memory information is set to reflect the fact that people’s memory is limited in real life. Factor $H_{x}$ adjusts the probability of individual choosing to imitate neighbor strategy in the next round by combining the characteristics of historical memory information and limited memory information. For the simplicity, $H_{x}$is calculated as follows:

2 $$\begin{aligned} H_{x} = {\left\{ \begin{array}{ll} &{} 1, \qquad \qquad \qquad \qquad M = 0 \\ &{} 1-(1-\beta )\cdot \frac{n_{x} }{M} ,\qquad \ \; M \ge 1 \end{array}\right. } \end{aligned}$$

Where, $\beta (0< \beta < 1)$ represents the influence of memory effect in the process of population evolution, that is, the weight of memory information *M* represents the length of each individual’s historical memory. $n_ {x}$ is the number of times an individual keeps continuous and the same as the current strategy within the length of *M* historical memory. Therefore, $H_{x}$ is always between 0 and 1. $n_ {x}$ = 0 indicates that the current strategy of the individual is different from that of the previous time, indicating that the individual is easy to change the strategy and is swinging. At this point, individual *i* will not be able to learn the strategy of individual *j*. It is worth noting that if an individual changes strategies many times in all the past rounds, the individual’s memory will be reset, which speeds up the process of population evolution, and the size of memory length *M* has become an important factor controlling cooperative evolution.

Learning mechanism: In the game process, player *x* randomly selects a neighbor *y* and utilizes Fermi rule^52^ to imitate its strategy with probability *P* from formula (3) : 

3 $$\begin{aligned} P(S_{x}\leftarrow S_{y})= \frac{H_{x}}{1+exp\frac{(P_{x}-P_{y})}{K}} \end{aligned}$$

Where $P_{x}$ represents the total payoff of individual *x*, $P_{y}$ represents the total payoff of individual *y*, and individual *x* and individual *y* obtain payoff in the same way in formula (3); *K* represents the uncertainty of strategy adoption during the game. *K* $\rightarrow$ 0 indicates that the player is absolutely rational in the decision-making process without noise interference; *K* $\rightarrow$ $\infty$ means that players are completely random in the decision-making process and are not affected by neighbor returns. According to the noise value used in previous studies, *K* is set 0.1.

## Data Availability

The datasets generated during and/or analysed during the current study are available from the corresponding author on reasonable request.
